# Comparing epidemiological and clinical data from RPS patients documented in a German cancer registry to a cohort from TARPSWG reference centres

**DOI:** 10.1007/s00432-024-06033-5

**Published:** 2024-11-28

**Authors:** Franziska Neemann, Lina Jansen, Silke Hermann, Christian Silcher, Madelaine Hettler, Peter Hohenberger, Dario Callegaro, Alessandro Gronchi, Marco Fiore, Rosalba Miceli, Frits Van Coevorden, Winan Van Houdt, Sylvie Bonvalot, Piotr Rutkowski, Jacek Skoczylas, Carol J. Swallow, Rebecca Gladdy, Dirk C. Strauss, Andrew Hayes, Mark Fairweather, Chandrajit P. Raut, Jens Jakob

**Affiliations:** 1 Department of Surgery, Sarcoma Unit, University Medical Center Mannheim (UMM) and Medical Faculty Mannheim, Heidelberg University, Theodor-Kutzer-Ufer 1-3, 68167 Mannheim, Germany; 2 Division of Surgical Oncology and Thoracic Surgery, University Medical Centre Mannheim (UMM) and Medical Faculty Mannheim, Heidelberg University, Mannheim, Germany; 3 German Cancer Research Center (DKFZ), Epidemiological Cancer Registry Baden-Württemberg, Heidelberg, Germany; 4 Sarcoma Service, Fondazione IRCCS Istituto Nazionale dei Tumori, Milan, Italy; 5 The Netherlands Cancer Institute, Amsterdam, The Netherlands; 6 Institut Curie, Paris, France; 7 Maria Sklodowska-Curie National Research Institute of Oncology, Warsaw, Poland; 8 Department of Surgery, Mount Sinai Hospital and Princess Margaret Cancer Centre University of Toronto, Toronto, Canada; 9 Royal Marsden Hospital NHS Foundation Trust, London, UK; 10 Brigham and Women’s Hospital, Dana-Farber Cancer Institute Harvard Medical School, Boston, USA; 11 Biostatistics for Clinical Research Unit, Fonazione IRCCS Istituto Nazionale dei Tumori, Milano, Italy

**Keywords:** Retroperitoneal sarcoma, Soft tissue sarcoma, Cancer registry, Healthcare related data, Certified cancer centre, Cohort study

## Abstract

**Purpose:**

Retroperitoneal sarcomas (RPS) are rare, heterogeneous tumours. Treatment recommendations are mainly derived from cohorts treated at reference centres. The applicability of data from cancer registries (CR) is controversial. This work compares CR and TARPSWG (Transatlantic Australasian Retroperitoneal Sarcoma Working Group) data to assess the representativeness of the TARPSWG and the applicability of the CR data.

**Methods:**

TARPSWG cohort has previously been described. The CR Baden-Württemberg cohort includes patients with primary RPS M0 (years 2016–2021, ICD-10 C.49.4/5, C48.x) who underwent surgery within 12 months. Only patients with sarcoma-typical histology codes as used for the German Cancer Society certification system were included. Patient, tumour and therapy factors as well as survival times were compared with Chi^2^-test, Kaplan Meier curves, and adjusted models.

**Results:**

1000 (TARPSWG) and 364 (CR) patients were included. CR patients were older (median: 64 years vs. 58 years), had more high-grade tumours (FNCLCC 3 48.1% vs. 27.4%, p < 0.0001) and the 5-year survival rate was significantly lower (56.3% vs. 67.9%, p = 0.0015). The proportions of dedifferentiated liposarcoma (CR 37.1% vs. 37.0%) and leiomyosarcoma (CR 20.1% vs. 19.2%), and patterns of recurrence in these most frequent RPS subtypes were similar.

**Conclusion:**

ICD-O/ICD 10 based filters appear to be a valid tool for extracting RPS cases from CR. The similar distribution and biological behavior of distinct RPS subtypes suggests that TARPS-WG are representative, and CR data may be used to verify recommendations derived from reference centre cohorts. Complementary use of data from different sources warrants further investigation in rare cancers.

**Supplementary Information:**

The online version contains supplementary material available at 10.1007/s00432-024-06033-5.

## Introduction

Soft tissue sarcomas are rare, solid tumors which arise from mesenchymal tissue and therefore can occur in almost every site of the body. With a crude incidence rate from 0.3 per 100.000 in Europe and North America, soft tissue sarcomas of the retroperitoneum count as rare cancers (Stiller et al. 2013; Porter et al. 2006; Mastrangelo et al. 2012; Ressing et al. 2018). The most frequent histological subtypes in RPS are Liposarcoma (LS), both well differentiated and dedifferentiated (WDLS and DDLS), and Leimyosarcoma (LMS) (Mack and Purgina 2022). The gold standard in therapy is macroscopically complete resection probably best achieved with a multivisceral resection (Leitlinienprogramm 2021). The potential role of neoadjuvant chemotherapy in high-risk tumours is under investigation (compare STRASS 2 Study, NCT04031677) The role of radiotherapy remains unclear for certain histotypes (Haas et al. 2019; Bonvalot et al. 2020; Callegaro et al. 2023); and recommendations vary between different centres and working groups (Rothermundt et al. 2023).

Currently, most data on the treatment and outcome of retroperitoneal sarcomas (RPS) come from retrospective studies of prospectively maintained databases in specialised centres; the majority of recommendations come from TARPSWG. Therefore, we selected one of the largest ever published RPS cohorts treated in reference centres for comparison. These data have been collected from eight well known reference centres who retrospectively analysed data which was prospectively maintained between 2002 and 2011 (Gronchi et al. 2016).

The German Cancer Registry Act (LKrebsRG) from 2011 has made it compulsory to report new cases of cancer (LKrebsRG. §4 (1) 2024). A standardized oncological basic data set is used for documentation. All healthcare providers involved in the diagnosis or treatment of cancer must report the following five items: diagnosis (ICD-10 Coding), pathology report (ICD-O Coding), specific cancer therapy (e.g. radiation therapy), disease progression or unremarkable follow-up or death. Information on vital status and cause of death are regularly obtained from residents’ registration offices. Standardization and mandatory participation are strengths of the registers. Weaknesses could arise from the completeness and quality of the reports (Katalinic et al. 2023).

Published reports from the cancer registry are usually presented according to localization expressed by ICD-10 coding. While this coding is practicable for common tumor entities such as breast or colon cancer, data evaluation for rarer tumors at different locations is challenging. We developed an extraction of RPS cases according to ICD-10 and ICD-O coding for this purpose. We wanted to compare epidemiological and oncological data of RPS patients from a CR cohort with a cohort from reference centers. If the cohorts were comparable in their composition, diagnostic and therapeutic strategies could be transferred from the reference centers to the general population. This is the first direct comparison of RPS patients between a population-based cohort and a cohort managed within specialized reference centers. The main question was whether conclusions reached based on patient outcomes from the latter source are transferable to general population.

## Methods

### Study population

The study is a retrospective statistical analysis of two cohorts. Data from the study of the Transatlantic Australasian Retroperitoneal Sarcoma Working Group (TARPSWG) served as the reference center cohort. The dataset includes patients treated for RPS at one of eight international centres between 2002 and 2011 and has been previously described (Gronchi et al. 2016). RPS patients who had no metastases at diagnosis (M0) and who underwent resection of the primary were included. Patients with GIST, Ewing Sarcoma, alveolar/ embryonal rhabdomyosarcoma, desmoid tumors and uterine sarcomas were excluded. From the TARPSWG cohort we additionally excluded patients with missing data (n = 7). The final cohort included 1000 patients.

**Fig. 1:**
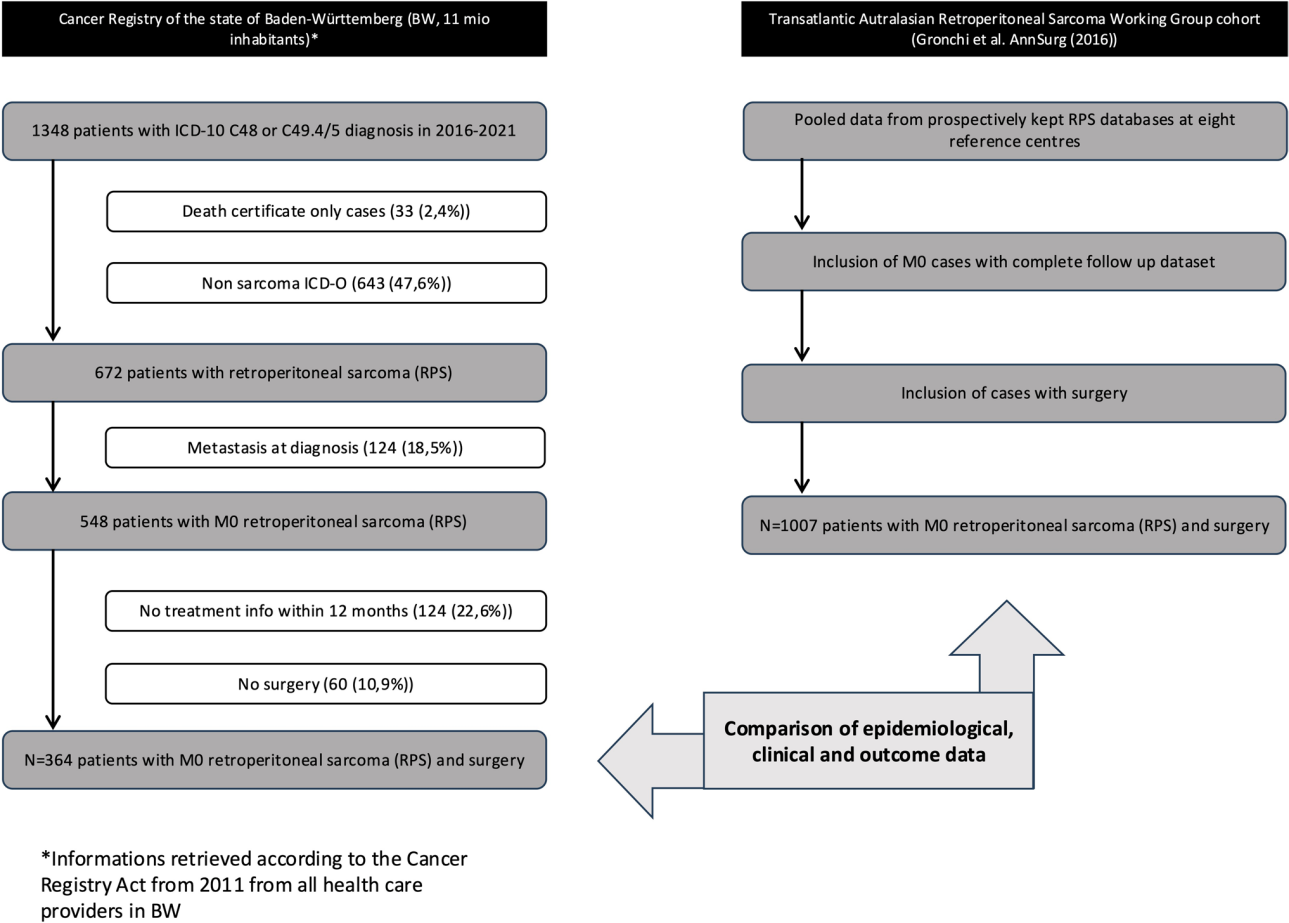
Study population and inclusion criteria in Cancer Registry cohort

Data from the population-based cancer registry (CR) of Baden-Württemberg served as a comparison dataset. The CR covers a population of 11.28 million people (2022) in the south-west of Germany. All patients aged 18 or older reported with RPS between 2016 and 2021 according to ICD-10 and ICD-O encoding were included. Patients who were only notified by death certificate to the cancer registry (DCO-cases (n = 33) and patients with non-RPS ICD-O (n = 643) were excluded (Fig. 1). Furthermore, we excluded all patients with metastasis at diagnosis (n = 124) and all patients without any treatment reported within 12 months (n = 124) and without surgery within 12 months (n = 60) of diagnosis. The final CR cohort included 364 patients (compare Fig. 1).

### Definition of covariates

In the TARPSWG cohort, data collection was planned a priori for RPS-specific analyses and, thus, detailed high-quality data was available. In the CR cohort, these factors had to be derived from the standard cancer registry data collection which e.g. does not focus on details of surgical procedures.

Age was categorized in five groups (18–44, 45–54, 55–64, 65–74 and 75 years or older). Seven histological subgroups were pre-defined in the TARPSWG cohort, and in the CR cohort these were identified by ICD-O-3 histological codes in the CR cohort: well differentiated liposarcoma (WDLS), dedifferentiated liposarcoma (DDLS), leiomyosarcoma (LMS), malignant peripheral-nerve sheath tumour (MPNST), undifferentiated pleomorphic sarcoma (UPS), solitary fibrous tumour (SFT) and other sarcomas (see Appendix). Grade as per Federation Nationale des Centres de Lutte Contre le Cancer (FNCLCC) system was directly recorded for the TARPSWG cohort, while Union Internationale Contre le Cancer (UICC) grade was recorded for the CR cohort. For the purpose of comparison here, it was inferred that UICC G1/2 equates with FNCLCC 1/2 and UICC G 3/4 equates with FNCLCC 3. Size was given as largest diameter (cm) in TARPSWG whereas in CR only T-stage was documented. The surgical margins were recorded as macroscopically complete (R0 and R1) or incomplete (R2) in both cohorts. Macroscopically complete margins tend to be an achievable result associated with improved overall survival and lower in both local and distant recurrence (Anaya et al. 2008). Chemo- and radiotherapy were directly recorded as pre and post operative in the TARPSWG cohort. For the CR cohort, the following definitions of neoadjuvant- and adjuvant-intent therapy were applied: for chemotherapy: 7 months pre surgery and 3 months post-surgery, for radiotherapy: 4 months pre surgery and 3 months post-surgery. It should be noted that the lack of any record of therapy according to the CR could reflect either that no treatment was administered at all, a treatment was administered outside of our definition time or that treatment was administered but not reported to the CR. Both cohorts include a vital status and recurrence follow-up and had information on cause of death (death due to sarcoma or other cause).

### Statistical methods

We compared the distributions of factors in the TARPSWG and CR cohort with Chi-square tests for categorical variables and Welch’s t-test for continuous variables. In addition, (multinomial) logistic regression models adjusted for sex and age (categorical) were computed.

In follow-up analyses, overall survival (OS) was defined as the time between date of diagnosis and death from any cause or last vital status report. In sarcoma-specific survival (SSS), only death due to sarcoma was counted as an event and patients who died from other causes were censored at date of death. For recurrence-free survival (RFS), first local recurrence, first distant metastasis or death from any cause (whichever occurred first) was counted as an event. Unadjusted OS, SSS, and RFS estimates were derived using the Kaplan–Meier method and compared with log-rank tests. Adjusted comparisons were computed using Cox proportional hazard models with adjustment for sex and age (categorical). The proportional hazard assumption was visually checked by plotting the log of the negative log of the survival function against time. It was statistically checked by adding time-dependent explanatory variables to the model.

Local recurrence (LR) and distant metastasis (DM) were additionally analysed in a competing-risk framework using crude cumulative incidence curves and Gray test for comparison across cohorts. Deaths without evidence of disease were counted as competing event. In the analysis of LR, DM were additionally counted as competing event. For DM, LRs were competing events. Concomitant LR and DM were included only in the analysis of DM.

Number of missing observations were reported in Tables and patients with missing information were excluded from the models. Statistical significance was defined with p < 0.05. All statistical analyses were conducted with SAS Enterprise Guide (Copyright© 2016 by SAS Institute Inc., Cary, NC, USA). Cumulative incidence curves were visualized using the %newsurv SAS Macro.

## Results

### Demographic data

Characteristics of the TARPSWG and CR cohort are shown in Table 1. While the gender distribution was comparable in both cohorts, strong age differences were observed with a median age of 64 years in the CR and 58 years in TARPS cohort (p < 0.0001). While 23.9% in the CR cohort were 75 years or older, the proportion was only 8.7% in the TAPRS cohort.

**Table 1:** Comparison of patient demographics, tumour characteristics and treatments received: comparison of CR and TARPSWG cohorts

Variable	Cancer Registry BW	TARPSWG	p Value^a^	Adj. comparison OR (95% CI), p Value^b^
Sex			0.0986	0.2900
Male	208 (57.1%)	521 (52.1%)		Ref
Female	156 (42.9%)	479 (47.9%)		0.87 (0.68–1.12)
Age at diagnosis			< 0.0001	< 0.0001
18–44	34 (9.3%)	188 (18.8%)		Ref
45–54	58 (15.9%)	231 (23.1%)		1.39 (0.87–2.21)
55–64	93 (25.5%)	268 (26.8%)		1.9 (1.23–2.93)
65–74	92 (25.3%)	226 (22.6%)		2.21 (1.42–3.42)
75 +	87 (23.9%)	87 (8.7%)		5.46 (3.41–8.74)
Median (IQR)	64 (20)	58 (19)	< 0.0001	
Grading			< 0.0001	< 0.0001
1	73 (23%)	326 (34.0%)		0.4 (0.29–0.55)
2	92 (28.9%)	370 (38.6%)		0.44 (0.32–0.6)
3	153 (48.1%)	263 (27.4%)		Ref
Missing	46	41		
Histology			< 0.0001	< 0.0001
WDLS	55 (15.1%)	261 (26.1%)		Ref: all LS
DDLS	135 (37.1%)	370 (37.0%)		
LS, grading missing	6 (1.6%)			
LMS	73 (20.1%)	192 (19.2%)		1.39 (1–1.94)
MPNST	3 (0.8%)	33 (3.3%)		0.49 (0.14–1.65)
SFT	2 (0.5%)	59 (5.9%)		0.13 (0.03–0.53)
Other	83 (22.8%)	63 (6.3%)		5.21 (3.51–7.72)
UPS	7 (1.9%)	22 (2.2%)		0.87 (0.36–2.15)
R status (first resection in CR)			0.6685	0.7608
R0/1	273 (94.8%)	954 (95.4%)		Ref
R2	15 (5.2%)	46 (4.6%)		1.1 (0.59–2.04)
RX or missing	76			
Chemotherapy (within timelimit)			0.2749	0.7175
No report/not done	307 (84.3%)	818 (81.8%)		Ref
Yes	57 (15.7%)	182 (18.2%)		1.06 (0.76–1.49)
Time: chemotherapy				Pre: 0.0053/post: < 0.0001
Preop (yes vs. no)	26 (7.1%)	151 (15.1%)	0.0001	0.53 (0.34–0.83)
Postop (yes vs. no)	34 (9.3%)	39 (3.9%)	0.0001	3.25 (1.98–5.33)
Radiotherapy (within timelimit)			0.1598	0.6208
No report/not done	262 (72%)	680 (68%)		Ref
Yes	102 (28%)	320 (32%)		0.93 (0.71–1.23)
Time: radiotherapy				P: 0.0065; 0.0087
Preop (yes vs. no)	51 (14%)	217 (21.7%)	0.0016	0.62 (0.44–0.88)
Postop (yes vs. no)	52 (14.3%)	109 (10.9%)	0.0865	1.64 (1.13–2.37)
Median follow-up (months)	44	58		

*BW* Baden-Württemberg, *OR* odds ratio, *CI* confidence interval, IQR interquartile range, *FNCLCC* Federation Nationale des Centres de Lutte Contre le Cancer, *WD* well differentiated, *DD* dedifferentiated, *LS* liposarcoma, *LMS* leiomyosarcoma, *MPNST* malignant peripheral-nerve sheath tumour, *UPS* undifferentiated pleomorphic sarcoma, *SFT* solitary fibrous tumour

^a^Chi-square test for categorical variables and Welch’s t-test for continuous variables

^b^(Multinomial) logistic regression model, adjusted for sex and age (categorical). Shown are the overall p value for the difference between studies for the respective factor and the odds ratio and 95% confidence interval for the level

### Tumour data

The CR cohort had fewer Grade 1/2 than TARPSWG (51.9% vs. 72.6%), and a correspondingly greater proportion of Grade 3 (48.1% than 27.4%). This difference remained significant after adjustment for age and sex (p < 0.0001). In terms of histological subtypes, the proportion of DDLS (37.1% CR vs. 37% TARPSWG) and LMS (20.1% vs. 19.2%) was comparable between both cohorts. By contrast, there were differences in the proportion of WDLS (15.1% vs. 26.1%), MPNST (0.8% vs. 3.3%), SFT (0.5% vs. 5.9%) and the group of “other” histological subtypes (22.8% vs. 6.3%). Histological distributions remained different after adjustment (p < 0.0001). The resection margin status was similar in both cohorts (adjusted p = 0.7608).

### Treatment data

There was no significant difference in the proportion of patients who received chemo- and/or radiation therapy between the two cohorts (adjusted comparison: p = 0.7175 for chemotherapy, p = 0.6208 for radiotherapy). With respect to the timing of therapy, planned preoperative (neoadjuvant) therapy was given less often in the CR cohort than the TARPSWG cohort (chemotherapy: 7.1% vs. 15.1%, p = 0.0001; radiotherapy: 14% vs. 21.7%, p = 0.0016). This pattern remained significant after adjustment for age and sex. Postoperative adjuvant chemotherapy was given more commonly in the CR cohort (9.3% vs. 3.9%, p = 0.0001). After adjustment, post-operative radiotherapy was also given more commonly in the CR cohort (p = 0.0087).

### Overall survival

**Fig. 2:**
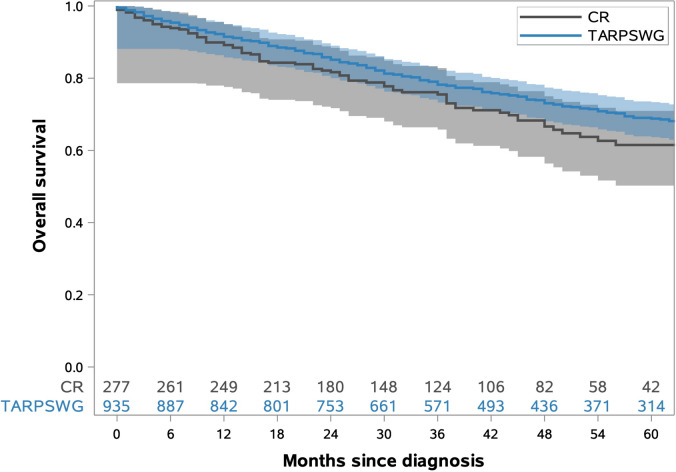
Overall survival curves CR vs. TARPSWG cohorts

As shown in Fig. 2a, overall survival (OS) at 5 years following resection was lower in the CR than in the TARPSWG cohort (56.3% vs. 67.9%, p = 0.0015). After adjustment for age, sex, histotype and r-status the hazard ratio (HR) was 0.99 (0.77–1.29).

### WDLS

Table 2 shows the comparison between patients with WDLS in CR vs. TARPSWG cohorts. In terms of grade, surgical margins, receipt of adjuvant RT and 5-year LR and DM rates the cohorts were comparable. In the CR cohort, age was on average 5 years older, and there was less frequent receipt of chemotherapy (1.8% vs. 5.7%) or radiotherapy (12.7% vs. 20.7%), in particular less neoadjuvant chemotherapy (0% vs. 5.4%) and/or neoadjuvant radiotherapy (7.3% vs. 15.7%). Age- and sex-adjusted analyses on treatment showed the same direction of the association but no significance and could sometimes not be conducted due to small case numbers. The 5-year OS for patients with WDLS was lower in the CR compared to the TARPSWG cohort (71.2 vs. 89.9%, HR 2.42).

**Table 2:** Patient, tumour, treatment, and outcome data for WDLS, DDLS and LMS, cancer registry vs. TARPSWG cohorts., CR vs. TARPSWG cohorts

Variables	Cancer Registry BW WDLS	TARPSWG WDLS	Adj. comparison^a^ (OR (95% CI), HR (95% CI), p-value)	Cancer Registry BW DDLS	TARPSWG DDLS	Adj. comparison^a^ (OR (95% CI), HR (95% CI), p-value)	Cancer Registry BW LMS	TARPSWG LMS	OR^a^ (95% CI), HR (95% CI), p-value
Sex									
Male	34 (61.8%)	145 (55.6%)	Ref	93 (68.9%)	213 (57.6%)	Ref	24 (32.9%)	70 (36.5%)	Ref
Female	21 (38.2%)	116 (44.4%)	0.82 (0.45–1.51)	42 (31.1%)	157 (42.4%)	0.64 (0.42–0.99)	49 (67.1%)	122 (63.5%)	1.22 (0.68–2.19)
Age at diagnosis									
18–54	15 (27.3%)	103 (39.5%)	Ref	26 (19.3%)	117 (31.6%)	Ref	18 (24.7%)	93 (48.4%)	Ref
55–74	28 (50.9%)	130 (49.8%)	1.43 (0.72–2.84)	76 (56.3%)	224 (60.5%)	1.44 (0.87–2.38)	41 (56.2%)	84 (43.8%)	2.52 (1.35–4.73)
75 +	12 (21.8%)	28 (10.7%)	2.87 (1.20–6.85)	33 (24.4%)	29 (7.8%)	4.89 (2.53–9.45)	14 (19.2%)	15 (7.8%)	4.90 (2.01–11.9)
Median (IQR)	64 (19)	59 (19)		66.0 (17.0)	60.0 (17.0)		65.0 (17.0)	55.0 (18.0)	
FNCLCC^b^									
1	52 (96.3%)	232 (93.2%)	Not estimable	3 (2.4%)	12 (3.4%)	0.49 (0.13–1.84)	8 (12.5%)	28 (14.6%)	0.96 (0.37–2.48)
2	1 (1.9%)	15 (6.0%)		44 (34.9%)	215 (60.4%)	0.33 (0.21–0.51)	32 (50%)	95 (49.5%)	0.94 (0.50–1.76)
3	1 (1.9%)	2 (0.8%)		79 (62.7%)	129 (36.2%)	Ref	24 (37.5%)	69 (35.9%)	Ref
Missing	1	12		30					
R status (first resection in CR)									
R0/1	41 (97.6%)	251 (96.2%)	Ref	102 (97.1%)	345 (93.2%)	Ref	60 (96.8%)	189 (98.4%)	Ref
R2	1 (2.4%)	10 (3.8%)	0.58 (0.07–4.76)	3 (2.9%)	25 (6.8%)	0.42 (0.12–1.43)	2 (3.2%)	3 (1.6%)	2.07 (0.30–14.08)
RX or missing	13	0					11		
Chemotherapy (within timelimit)^c^									
No report/not done	54 (98.2%)	246 (94.3%)	Ref	112 (83%)	306 (82.7%)	Ref	64 (87.7%)	137 (71.4%)	Ref
Yes	1 (1.8%)	15 (5.7%)	0.33 (0.04–2.59)	23 (17%)	64 (17.3%)	1.16 (0.67–1.99)	9 (12.3%)	55 (28.6%)	0.41 (0.19–0.91)
Time: chemotherapy									
Preop (yes vs. no)	0	14 (5.4%)	Not estimable	9 (6.7%)	53 (14.3%)	0.49 (0.23–1.04)	5 (6.8%)	45 (23.4%)	0.28 (0.10–0.75)
Postop (yes vs. no)	1 (1.8%)	2 (0.8%)	Not estimable	14 (10.4%)	11 (3%)	4.36 (1.88–10.1)	4 (5.5%)	13 (6.8%)	0.94 (0.29–3.08)
Radiotherapy (within timelimit)									
No report/not done	48 (87.3%)	207 (79.3%)	Ref	89 (65.9%)	245 (66.2%)	Ref	55 (75.3%)	120 (62.5%)	Ref
Yes	7 (12.7%)	54 (20.7%)	0.67 (0.28–1.62)	46 (34.1%)	125 (33.8%)	1.02 (0.66–1.56)	18 (24.7%)	72 (37.5%)	0.54 (0.29–1.01)
Time: radiotherapy									
Preop (yes vs. No)	4 (7.3%)	41 (15.7%)		25 (18.5%)	87 (23.5%)	0.63 (0.38–1.07)	7 (9.6%)	53 (27.6%)	0.29 (0.12–0.68)
Postop (yes vs. No)	3 (5.5%)	15 (5.7%)	Not estimable	21 (15.6%)	38 (10.3%)	2.05 (1.13–3.71)	11 (15.1%)	21 (10.9%)	1.42 (0.63–3.21)
LR (2 years)									
No report/not done	51 (92.7%)	244 (93.5%)		91 (67.4%)	269 (72.7%)		63 (86.3%)	170 (88.5%)	
Yes	4 (7.3%)	17 (6.5%)	1.02 (0.32–3.24)	44 (32.6%)	101 (27.3%)	1.34 (0.86–2.09)	10 (13.7%)	22 (11.5%)	1.44 (0.62–3.30)
DM (2 years)									
No report/not done	54 (98.2%)	259 (99.2%)		118 (87.4%)	321 (86.8%)		56 (76.7%)	129 (67.2%)	
Yes	1 (1.8%)	2 (0.8%)	3.11 (0.23–42.9)	17 (12.6%)	49 (13.2%)	0.92 (0.5–1.69)	17 (23.3%)	63 (32.8%)	0.70 (0.37–1.32)
OS 1-year	94.2 (83.2–98.1)	96.9 (93.9–98.5)	1.00 (0.26–3.8)	87.4 (80.5–92)	88.5 (84.8–91.4)	0.90 (0.51–1.58)	93.2 (84.3–97.1)	93.1 (88.4–95.9)	1.07 (0.39–2.91)
OS 5-years	71.2 (53.6–83.1)	89.9 (84.5–93.4)	2.42 (1.17–5.02)	56.6 (44.8–66.7)	57 (51–62.6)	0.88 (0.62–1.25)	57.6 (38.8–72.5)	62.3 (53.9–69.7)	1.00 (0.58–1.72)
CS 1-year	96 (84.8–99)	98.8 (96.5–99.6)	2.39 (0.38–14.94)	87.7 (80.7–92.3)	91.4 (88–93.9)	1.14 (0.62–2.11)	93.1 (84.1–97)	95.7 (91.6–97.8)	1.54 (0.51–4.63)
CS 5-years	80.2 (63.4–89.9)	95.6 (91.2–97.8)	4.59 (1.73–12.15)	64.3 (51.9–74.3)	61.5 (55.3–67)	0.86 (0.58–1.27)	58.8 (39.6–73.8)	66.5 (57.9–73.8)	1.20 (0.67–2.14)
LR 1-year	7.5 (2.4–16.7)	1.9 (0.7–4.2)	3.46 (1.07–11.21)	19.3 (13.1–26.3)	11.7 (8.7–15.3)	1.69 (1.04–2.74)	6.8 (2.5–14.2)	2.6 (1.00–5.7)	2.76 (0.82–9.25)
LR 5-years	23.7 (11.5–38.3)	23.6 (17.7–30)	1.17 (0.59–2.32)	44.1 (33.9–53.7)	39 (33.6–44.4)	1.27 (0.91–1.77)	12.2 (5.5–21.8)	9.9 (5.8–15.3)	1.65 (0.68–3.99)
DM 1-year	0.0	0.0	N/A	9.6 (5.4–15.3)	8.2 (5.7–11.3)	1.11 (0.58–2.12)	15.1 (8–24.3)	19.5 (14.2–25.4)	0.84 (0.41–1.71)
DM 5-years	0.0	0.4 (0–2.1)	N/A	13.4 (8.1–20.1)	18.3 (14.3–22.7)	0.74 (0.44–1.26)	53.2 (29.6–72.1)	50.5 (42.5–57.9)	0.81 (0.52–1.26)

*OR* odd ratio, *CI* confidence interval, *HR* hazard ratio, *ref* reference, *IQR* interquartile range, *FNCLCC* Federation Nationale des Centres de Lutte Contre le Cancer, *LR* Local recurrence, *DM* distant Metastasis, *N/A* not estimable due to small case numbers, *OS* overall survival, *CS* cause-specific survival, *CIF* cumulative incidence function

^a^For frequencies: (multinomial) logistic regression model, adjusted for sex and age (categorical). For survival: Cox proportional hazard model restricted to the length of follow-up. Shown are the overall p values for the difference between cohorts (reference: TARPSWG) for the respective factor and the odds ratio/hazard ratio and 95% confidence interval for the level

^b^FNCLCLL grading estimated as described in methods

^c^Time limits as described in methods

### DDLS

**Fig. 3:**
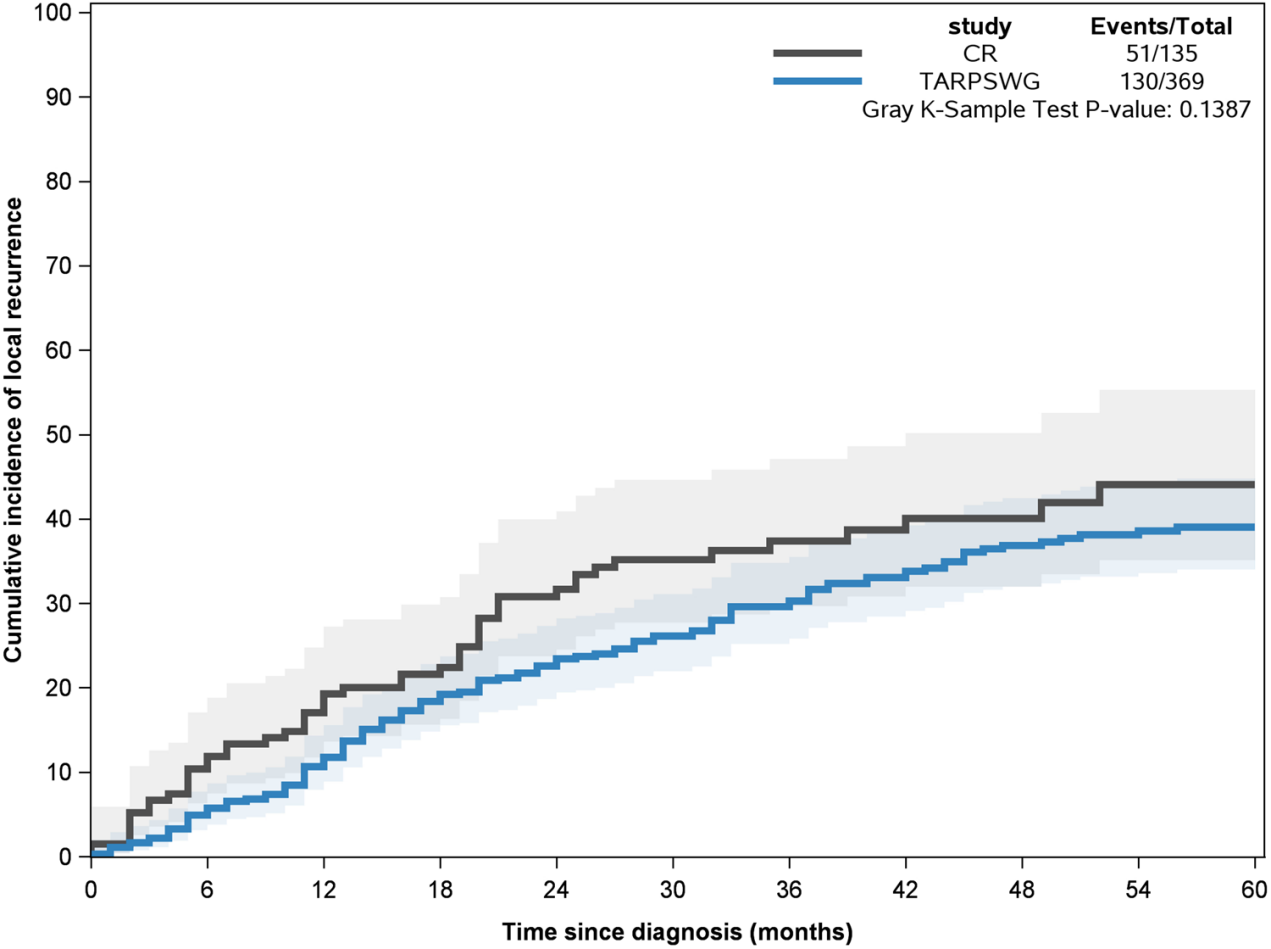
Crude cumulative incidence of local recurrence in patients with DDLS, CR vs. TARPS cohorts

Compared to the TARPSWG cohort, patients with DDLS in the CR cohort were less often female (31.1% vs. 42.4%, adjusted OR: 0.64), were on average six years older, had less often grade 2 (34.9% vs. 60.4%, OR: 0.33) but more often grade 3 and had less often an R2 status (2.9% vs. 6.8%, OR: 0.42, Table 2). While receipt of chemo- and radiotherapy were overall comparable in both cohorts, the setting was more often post-surgery in the CR cohort (chemotherapy: 10.4% vs. 3%, OR 4.38; radiotherapy: 15.6% vs. 10.3%; OR 2.05). After adjustment for sex and age, there was a tendency to higher 5-year OS (0.88) and CS (0.86) rates for patients in the CR. Figure 3 shows the crude cumulative incidence function of LR. The incidence of LR is higher in the CR cohort after 1 year, even after adjustment for sex and age (HR 1.69), but comparable after 5-years.

### LMS

**Fig. 4:**
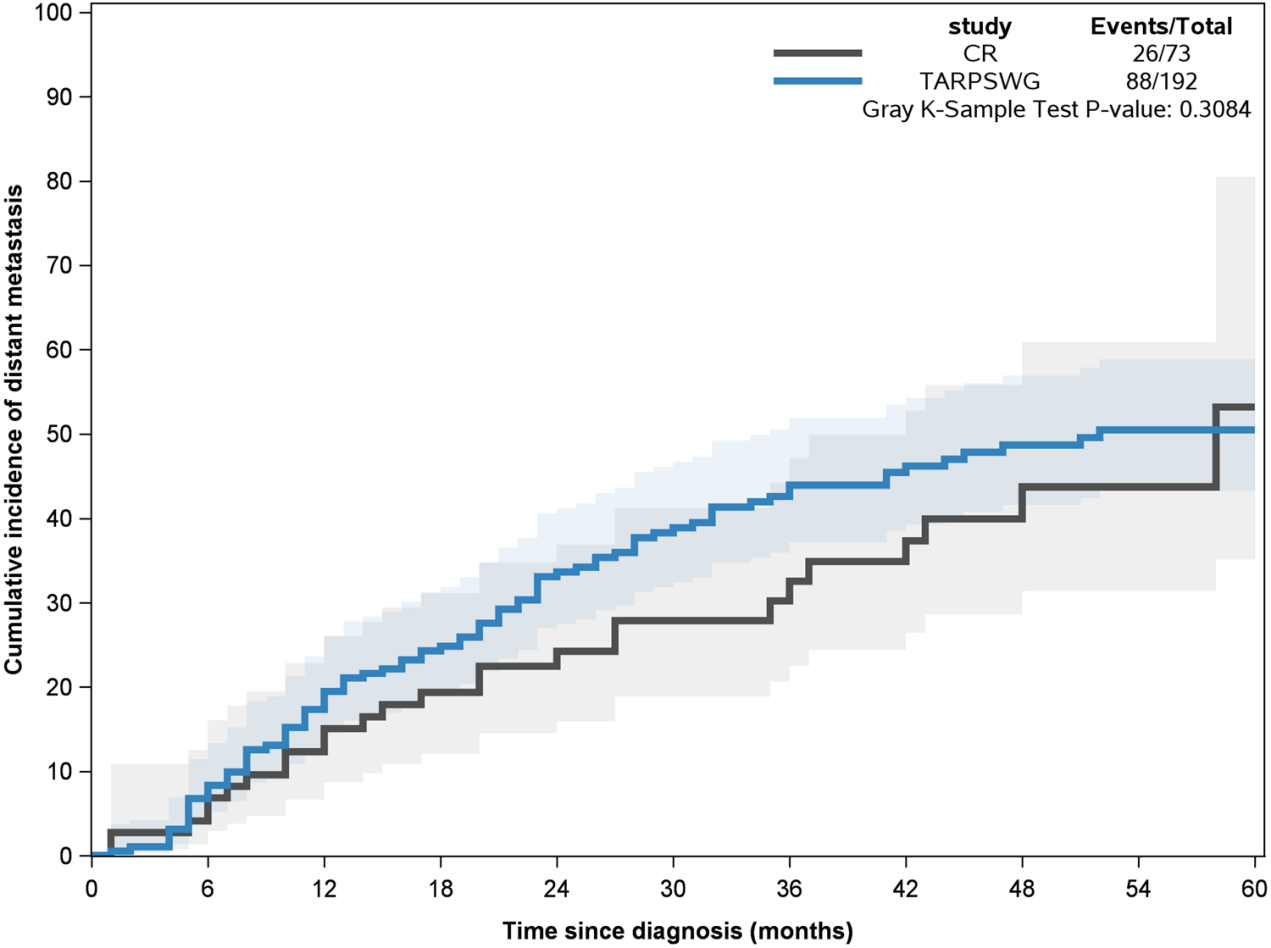
Crude cumulative incidence of distant metastasis in patients with LMS

Table 2 shows the comparison between patients with LMS in the CR vs. TARPSWG cohorts. In terms of sex, grade, surgical margins and 5-year LR rate, the cohorts were comparable. In the CR cohort, age was on average 10 years older, and patients were less likely to receive chemotherapy (12.3% vs. 28.6%) and/or radiotherapy (24.7% vs. 37.5%), particularly neoadjuvant chemotherapy (6.8% vs. 23.4%) and/or radiotherapy (9.6% vs. 27.6%), than in the TARPSWG cohort. After adjustment for sex and age, 5-year OS, CS, and cumulative incidence of local recurrences were comparable. While there was overall no significant difference in the cumulative incidence of distant metastasis within 5 years (crude: p = 0.3084, Fig. 4, adjusted HR: 0.81), five to 58 months following resection the incidence was slightly lower in the CR than the TARPSWG cohort.

## Discussion

This is the first direct comparison of RPS patients within a population-based cohort to a cohort managed within specialised reference centres. The main question was whether conclusions reached based on patient outcomes from the latter source are transferable to general population.

Regarding tumour and patient characteristics, there were relevant differences in age, RPS grade, proportion of patients with WDLS, and fraction of “other histological subtypes”. Sarcomas are tumours with an age-specific incidence rising with age and an average age at diagnosis varying from 57 to 67 years (LKrebsRG. §4 (1) 2024). Although age distribution was different, the median age of the TARPSWG cohort and the CR cohort was within this range. We assume a selection bias with younger patients treated at reference centres.

Both cohorts follow the classic histological distribution for RPS, with DDLS being the most common subtype, followed by WDLS and LMS. MPNST, SFT and UPS form smaller groups which differ in rank between different studies (Raut et al. 2016; Fletcher et al. 2013). There was a larger fraction of high-grade RPS in CR compared to TARPSWG. These differences may be explained by an uncertainty in histologic evaluation at the various centers that contributed to CR and the different ways that grading is being done across centres and countries. A meaningful comparison of the tumor size was not feasible. Tumor size was only entered as T-stage in the CR. Until the last amendment, this only provided for a differentiation of tumours smaller or larger than 5 cm and was of no significance for RPS. This shows a major disadvantage of the standardized data structure of cancer registries compared to specialized entity-specific registries.

The gold standard for resection of RPS is complete en-bloc resection. For LS, a multivisceral resection seems to provide best outcome while in other histotypes a more restrictive resection may be achievable (Bonvalot et al. 2023). This tailor-made strategy was developed in high-volume reference centres and is well documented in TARPSWG. A comparison of surgical strategies with CR was not feasible since extend of resection was not documented and only resection margins are given. However, the fraction of patients with R2 resection, and therefore the proportion of tumours where complete resection was not achievable, was 5% in both cohorts. Data on case volume were not available in the CR. Both reference centers as well as hospitals that only operate RPS sporadically contributed to the CR.

The proportion of applied chemo and/or radiotherapy were overlapping while we observe a higher proportion of adjuvant treatment in CR other than TARPSWG which prefers neoadjuvant treatment. TARPSWG cohort profits from treatment in reference centers which is known to provide the best guideline compliant care including throughout pretreatment biopsy and imaging (Bonvalot et al. 2019). Biopsy and Imaging are a prerequisite für tailored surgery. We may assume that in non-reference centers, which also contributed to CR, this preparation was not made and the decision on the resection strategy suffers from this (Jakob et al. 2018). To date recommendations to treat RPS with chemotherapy or radiation therapy often favor a neoadjuvant approach rather than an adjuvant one, depending on grading and subtype (Bree et al. 2023).

OS was generally better in TARPSWG cohort than in CR cohort. As seen in Fig. 2b the adjusted analysis for OS no longer showed a difference between both cohorts. An underlying disruptive factor may be the larger proportion of patients with WDLS in the TARPSWG cohort. This could contribute to the longer OS of the TARPSWG cohort due to longer OS of WDLS patients in general (Singer et al. 2003). On the other hand, treatment in reference centres could have led to the better OS due to multimodal treatment and extended, histology-tailored surgery (Bonvalot et al. 2009). Data from entity-specific registries in France clearly indicate a survival advantage from treatment (surgery) at specialised centres (Bonvalot et al. 2019). Beside better survival, we would have expected fewer relapses in the TARPSWG cohort which was not the case. One reason may be that while link of CRs to population registries allows a complete analysis of survival, random samples of centre and CR data cannot rule out underreporting of recurrences.

The strength of the study is that we were able to identify the most frequent RPS subtypes (DDLS and LMS) in the CR by applying a data filter based on ICD 10 and ICD-O codes. The differentiation and identification of sarcoma subtypes according to localisation and histology is of utmost importance as they exhibit distinct patterns of recurrence resulting in different recommendations for surgical, radiotherapeutic and systemic treatment (Leitlinienprogramm 2021; Swallow et al. 2021; Gronchi et al. 2021). Patients with retroperitoneal LMS develop more frequently metastases and less frequently local recurrences than patients with retroperitoneal DDLS and vice versa (Gronchi et al. 2016). We achieved a reliable identification of RPS subtypes by using an ICD-O filter comprising those histological subtypes that were proposed by expert sarcoma pathologists for case definition in the context of the certification process for specialised sarcoma centres in Germany. The resulting CR cohorts of DDLS and LMS patients revealed no significant differences in terms of CCI of LR and DM. The similarity of these typical oncological events demonstrates the similarity of the cohorts since they reflect a typical pattern of tumour biology.

Randomised trials are the gold standard in clinical research, including in rare cancers. Due to the low incidence and their heterogeneity, randomised studies in sarcomas are a major challenge. Cancer registry data comprise the largest possible number of patients, but the data structure is usually adapted to the more common (carcinoma) entities. A differentiated evaluation analogous to prospective studies or specialised entity registries (e.g. for retroperitoneal DDLS or LMS) is frequently not feasible. A combination of localisation- and histology-based filters, supported by comparisons with entity-specific registries, could represent an effective tool in cancer registry research.

The complementary use of different data sources may improve the assessment of therapeutic interventions using cancer registry data to generate more evidence in questions of controversial debate. As an example, the randomized STRASS 1 trial failed to demonstrate an improvement in abdominal recurrence-free survival through preoperative radiotherapy in RPS in general (Bonvalot et al. 2020). Proponents of radiotherapy analysed a cohort of RPS patients who had not taken part in the study but were treated at the same reference centres during the same period (the so-called STREXIT cohort) demonstrating a benefit for the subgroup of retroperitoneal liposarcoma (Callegaro et al. 2023). A cohort of carefully selected CR cases could be suitable to re-evaluate STRASS 1 and STREXIT to test whether a benefit of preoperative RT in retroperitoneal liposarcoma patients can be seen in the general population. However, an effective comparison may require the integration of additional items into the CR [e.g. derived from established nomograms (Callegaro et al. 2024)], as well as an effort in amending the under-reporting of treatment and disease progression. Ideally, a data link between data from cancer registries, reference centre cohorts and other sources such as health insurance data could be established (Bobeth et al. 2023).

## Study limitations

The study has several limitations. The retrospective design carries the risk of selection bias and data loss. Another limitation is the smaller number of patients in the CR cohort and the different time of data collection (6 years for CR vs. 10 years for TARPSWG). In addition, the selected TARPSWG cohort might be less updated compared to the current CR data set. Nevertheless, we decided TARPSWG as a reference since data were available, published and validated. Moreover, TARPSWG has developed a distinct RPS specific dataset to evaluate treatment and outcome of RPS patients. In contrast, the CR was developed as a general cancer registry that leaves little possibilities for the documentation of entity-specific parameters.

## Conclusions

In conclusion, the present study suggests that filters based on ICD 10 and ICD-O codes are an effective tool to extract sarcoma cases from cancer registries. The differences between the CR and the TARPSWG cohorts such as age and OS can be explained by the ‘‘reference centre effect’’ while the differences in grading and histology can be explained by possible underreporting and missing reference pathologies. Despite these differences in the overall cohorts, the similar proportions and patterns of recurrence of the most frequent subtypes (DDLS and LMS) suggest that TARPSWG data are representative when tumour and patient characteristics are considered. Recommendations for specific treatment settings and histological subgroups derived from the reference centres may therefore be transferred to the general population. At the same time, carefully selected cases from cancer registries offer the opportunity to review these recommendations, especially if they are the subject of controversial debate. Linking data from these and other sources may create synergies in the future.

## Supplementary Information

Below is the link to the electronic supplementary material.

Supplementary file1 (PDF 91 KB)

## Data Availability

No datasets were generated or analysed during the current study.

## References

[CR1] Anaya DA, Lev DC, Pollock RE (2008) The role of surgical margin status in retroperitoneal sarcoma. J Surg Oncol 98(8):607–610. 10.1002/jso.21031 19072853 10.1002/jso.21031

[CR2] Bobeth C, Tol KK, Rossler M, Bierbaum V, Gerken M, Gunster C et al (2023) Methodology and attribution success of a data linkage of clinical registry data with health insurance data. Gesundheitswesen 85(S 02):S154–S161. 10.1055/a-1984-0085 36940697 10.1055/a-1984-0085

[CR3] Bonvalot S, Rivoire M, Castaing M, Stoeckle E, Le Cesne A, Blay JY et al (2009) Primary retroperitoneal sarcomas: a multivariate analysis of surgical factors associated with local control. J Clin Oncol 27(1):31–37. 10.1200/JCO.2008.18.0802 19047280 10.1200/JCO.2008.18.0802

[CR4] Bonvalot S, Gaignard E, Stoeckle E, Meeus P, Decanter G, Carrere S et al (2019) Survival benefit of the surgical management of retroperitoneal sarcoma in a reference center: a nationwide study of the French Sarcoma Group from the NetSarc Database. Ann Surg Oncol 26(7):2286–2293. 10.1245/s10434-019-07421-9 31065964 10.1245/s10434-019-07421-9

[CR5] Bonvalot S, Gronchi A, Le Péchoux C, Swallow CJ, Strauss D, Meeus P et al (2020) Preoperative radiotherapy plus surgery versus surgery alone for patients with primary retroperitoneal sarcoma (EORTC-62092: STRASS): a multicentre, open-label, randomised, phase 3 trial. Lancet Oncol 21(10):1366–1377. 10.1016/s1470-2045(20)30446-0 32941794 10.1016/S1470-2045(20)30446-0

[CR6] Bonvalot S, Gronchi A, Le Pechoux C, Swallow CJ, Strauss D, Meeus P et al (2020) Preoperative radiotherapy plus surgery versus surgery alone for patients with primary retroperitoneal sarcoma (EORTC-62092: STRASS): a multicentre, open-label, randomised, phase 3 trial. Lancet Oncol 21(10):1366–1377. 10.1016/S1470-2045(20)30446-0 32941794 10.1016/S1470-2045(20)30446-0

[CR7] Bonvalot S, Roland C, Raut C, Le Pechoux C, Tzanis D, Frezza AM et al (2023) Histology-tailored multidisciplinary management of primary retroperitoneal sarcomas. Eur J Surg Oncol 49(6):1061–1067. 10.1016/j.ejso.2022.05.010 35599138 10.1016/j.ejso.2022.05.010

[CR8] Callegaro D, Raut CP, Ajayi T, Strauss D, Bonvalot S, Ng D et al (2023) Preoperative radiotherapy in patients with primary retroperitoneal sarcoma: EORTC-62092 trial (STRASS) versus off-trial (STREXIT) results. Ann Surg 278(1):127–134. 10.1097/SLA.0000000000005492 35833413 10.1097/SLA.0000000000005492

[CR9] Callegaro D, Barretta F, Raut CP, Johnston W, Strauss DC, Honore C et al (2024) New sarculator prognostic nomograms for patients with primary retroperitoneal sarcoma: case volume does matter. Ann Surg 279(5):857–865. 10.1097/SLA.0000000000006098 37753660 10.1097/SLA.0000000000006098

[CR10] de Bree E, Michelakis D, Heretis I, Kontopodis N, Spanakis K, Lagoudaki E et al (2023) Retroperitoneal soft tissue sarcoma: emerging therapeutic strategies. Cancers (Basel). 10.3390/cancers15225469 38001729 10.3390/cancers15225469 PMC10670057

[CR11] Fletcher CDMBJ, Bridge JA, Hogendoorn PCW, Mertens F (2013) Pathology and genetics of tumours of soft tissue and bone. WHO classification of tumours of soft tissue and bone, 4th edn. IARC Press, Lyon

[CR12] Gronchi A, Strauss DC, Miceli R, Bonvalot S, Swallow CJ, Hohenberger P et al (2016) Variability in patterns of recurrence after resection of primary retroperitoneal sarcoma (RPS): a report on 1007 patients from the multi-institutional collaborative RPS Working Group. Ann Surg 263(5):1002–1009. 10.1097/SLA.0000000000001447 26727100 10.1097/SLA.0000000000001447

[CR13] Gronchi A, Miah AB, Dei Tos AP, Abecassis N, Bajpai J, Bauer S et al (2021) Soft tissue and visceral sarcomas: ESMO-EURACAN-GENTURIS clinical practice guidelines for diagnosis, treatment and follow-up(☆). Ann Oncol 32(11):1348–1365. 10.1016/j.annonc.2021.07.006 34303806 10.1016/j.annonc.2021.07.006

[CR14] Haas RLM, Bonvalot S, Miceli R, Strauss DC, Swallow CJ, Hohenberger P et al (2019) Radiotherapy for retroperitoneal liposarcoma: a report from the Transatlantic Retroperitoneal Sarcoma Working Group. Cancer 125(8):1290–1300. 10.1002/cncr.31927 30602058 10.1002/cncr.31927 PMC6590287

[CR15] Jakob J, Gerres A, Ronellenfitsch U, Pilz L, Wartenberg M, Kasper B et al (2018) Treatment of retroperitoneal sarcoma in Germany: Results of a survey of the German Society of General and Visceral Surgery, the German Interdisciplinary Sarcoma Study Group and the advocacy group Das Lebenshaus. Chirurg 89(1):50–55. 10.1007/s00104-017-0504-2 28905080 10.1007/s00104-017-0504-2

[CR16] Katalinic A, Halber M, Meyer M, Pfluger M, Eberle A, Nennecke A et al (2023) Population-based clinical cancer registration in Germany. Cancers (Basel). 10.3390/cancers15153934 37568750 10.3390/cancers15153934 PMC10416989

[CR17] Leitlinienprogramm (2021). https://www.leitlinienprogramm-onkologie.de/fileadmin/user_upload/Downloads/Leitlinien/Adulte_Weichgewebesarkome/LL_Weichgewebesarkome_Langversion_1.1.pdf.

[CR18] LKrebsRG. §4 (1) (2024)

[CR19] Mack T, Purgina B (2022) Updates in pathology for retroperitoneal soft tissue sarcoma. Curr Oncol 29(9):6400–6418. 10.3390/curroncol29090504 36135073 10.3390/curroncol29090504 PMC9497884

[CR20] Mastrangelo G, Coindre JM, Ducimetiere F, Dei Tos AP, Fadda E, Blay JY et al (2012) Incidence of soft tissue sarcoma and beyond: a population-based prospective study in 3 European regions. Cancer 118(21):5339–5348. 10.1002/cncr.27555 22517534 10.1002/cncr.27555

[CR21] Porter GA, Baxter NN, Pisters PW (2006) Retroperitoneal sarcoma: a population-based analysis of epidemiology, surgery, and radiotherapy. Cancer 106(7):1610–1616. 10.1002/cncr.21761 16518798 10.1002/cncr.21761

[CR22] Raut CP, Miceli R, Strauss DC, Swallow CJ, Hohenberger P, van Coevorden F et al (2016) External validation of a multi-institutional retroperitoneal sarcoma nomogram. Cancer 122(9):1417–1424. 10.1002/cncr.29931 26916507 10.1002/cncr.29931

[CR23] Ressing M, Wardelmann E, Hohenberger P, Jakob J, Kasper B, Emrich K et al (2018) Strengthening health data on a rare and heterogeneous disease: sarcoma incidence and histological subtypes in Germany. BMC Public Health 18(1):235. 10.1186/s12889-018-5131-4 29433465 10.1186/s12889-018-5131-4 PMC5809940

[CR24] Rothermundt C, Andreou D, Blay JY, Brodowicz T, Desar IME, Dileo P et al (2023) Controversies in the management of patients with soft tissue sarcoma: recommendations of the conference on state of science in sarcoma 2022. Eur J Cancer 180:158–179. 10.1016/j.ejca.2022.11.008 36599184 10.1016/j.ejca.2022.11.008

[CR25] Singer S, Antonescu CR, Riedel E, Brennan MF (2003) Histologic subtype and margin of resection predict pattern of recurrence and survival for retroperitoneal liposarcoma. Ann Surg 238(3):358–370. 10.1097/01.sla.0000086542.11899.38. (**discussion 70–1**) 14501502 10.1097/01.sla.0000086542.11899.38 PMC1422708

[CR26] Stiller CA, Trama A, Serraino D, Rossi S, Navarro C, Chirlaque MD et al (2013) Descriptive epidemiology of sarcomas in Europe: report from the RARECARE project. Eur J Cancer 49(3):684–695. 10.1016/j.ejca.2012.09.011 23079473 10.1016/j.ejca.2012.09.011

[CR27] Swallow CJ, Strauss DC, Bonvalot S, Rutkowski P, Desai A, Gladdy RA et al (2021) Management of primary retroperitoneal sarcoma (RPS) in the adult: an updated consensus approach from the Transatlantic Australasian RPS Working Group. Ann Surg Oncol 28(12):7873–7888. 10.1245/s10434-021-09654-z 33852100 10.1245/s10434-021-09654-z PMC9257997

