# Supplementary material for: Comparing epidemiological and clinical data from RPS patients documented in a German cancer registry to a cohort from TARPSWG reference centres
Source: J Cancer Res Clin Oncol. 2024 Nov 28;150(12):514. doi: 10.1007/s00432-024-06033-5 (PMC11602858; doi:10.1007/s00432-024-06033-5)
Supplement: Supplementary file 1 — Supplementary file1 (PDF 91 KB) [file 432_2024_6033_MOESM1_ESM.pdf]

Appendix

ICD - O Code	Tumour	Frequency d	Percentage
8800/3	Sarcoma NOS	7	8,43
8801/3	Spindle-cell sarcoma	7	8,43
8802/3	Giant cell sarcoma	18	21,69
8804/3	Epitheloid sarcoma	3	3,61
8810/3	Fibrosarcoma	6	7,23
8811/3	Myofibrosarcoma	6	7,23
8813/3	Fascial fibrosarcoma	1	1,20
8825/3	Myofibroblastic sarcoma	2	2,41
8840/3	Myxosarcoma	1	1,20
8852/3	Myxoid LS	12	14,46
8900/3	Rhabdomyosarcoma	2	2,41
8901/3	Adult pleomorphic rhabdomyosarcoma	2	2,41
8912/3	Spindle-cell rhabdomyosarcoma	2	2,41
9040/3	Synovial sarcoma	5	6,02
9043/3	Biphasic Synovial sarcoma	1	1,20
9120/3	Haemangiosarcoma	7	8,43
9133/3	Epithelioid Haemangioendotheloim	1	1,20

Appendix Table 1. ***ICD O Code of ''other'' Histologies in CR***

ICD - O Code	Tumour	Histology Group
8851/3	well diff. LS	WDLS
8854/3	pleomorphic LS	DDLS
8858/3	dediff. LS	DDLS
8890/3	LMS	LMS
8891/3	Epithelioid LMS	LMS
9540/3	MPNST	MPNST
8815/3	SFT	SFT
8805/3	undiff. pleomorphic Sarcoma	UPS
8830/3	mal. fibrous cytoma	UPS

Appendix Table 2. ***ICD O Code classification into Subgroups***
